# AMAZONE: prevention of persistent pain after breast cancer treatment by online cognitive behavioral therapy—study protocol of a randomized controlled multicenter trial

**DOI:** 10.1186/s13063-022-06549-6

**Published:** 2022-07-25

**Authors:** Anne Lukas, Maurice Theunissen, Dianne de Korte-de Boer, Sander van Kuijk, Lotte Van Noyen, Walter Magerl, Werner Mess, Wolfgang Buhre, Madelon Peters

**Affiliations:** 1 Department of Anesthesiology & Pain Medicine, Maastricht University Medical Center+, Maastricht, The Netherlands; 2 Department of Clinical Psychological Science, Maastricht University, Maastricht, The Netherlands; 3 Department of Clinical Epidemiology and Medical Technology, Maastricht University Medical Center+, Maastricht, The Netherlands; 4 Department of Neurophysiology, Mannheim Center for Translational Neuroscience (MCTN), Ruprecht-Karls-University Heidelberg, Medical Faculty Mannheim, Heidelberg, Germany; 5 Department of Clinical Neurophysiology, Maastricht University Medical Center+, Maastricht, The Netherlands

**Keywords:** Persistent postoperative pain, Breast cancer, Survivorship, Prevention, Cognitive behavioral therapy, Psychological risk factors, Online, RCT, QST, CPM

## Abstract

**Background:**

Surviving breast cancer does not necessarily mean complete recovery to a premorbid state of health. Among the multiple psychological and somatic symptoms that reduce the quality of life of breast cancer survivors, persistent pain after breast cancer treatment (PPBCT) with a prevalence of 15–65% is probably the most invalidating. Once chronic, PPBCT is difficult to treat and requires an individualized multidisciplinary approach. In the past decades, several somatic and psychological risk factors for PPBCT have been identified. Studies aiming to prevent PPBCT by reducing perioperative pain intensity have not yet shown a significant reduction of PPBCT prevalence. Only few studies have been performed to modify psychological distress around breast cancer surgery. The AMAZONE study aims to investigate the effect of online cognitive behavioral therapy (e-CBT) on the prevalence of PPBCT.

**Methods:**

The AMAZONE study is a multicenter randomized controlled trial, with an additional control arm. Patients (*n*=138) scheduled for unilateral breast cancer surgery scoring high for surgical or cancer-related fears, general anxiety or pain catastrophizing are randomized to receive either five sessions of e-CBT or online education consisting of information about surgery and a healthy lifestyle (EDU). The first session is scheduled before surgery. In addition to the online sessions, patients have three online appointments with a psychotherapist. Patients with low anxiety or catastrophizing scores (*n*=322) receive treatment as usual (TAU, additional control arm).

Primary endpoint is PPBCT prevalence 6 months after surgery.

Secondary endpoints are PPBCT intensity, the intensity of acute postoperative pain during the first week after surgery, cessation of postoperative opioid use, PPBCT prevalence at 12 months, pain interference, the sensitivity of the nociceptive and non-nociceptive somatosensory system as measured by quantitative sensory testing (QST), the efficiency of endogenous pain modulation assessed by conditioned pain modulation (CPM) and quality of life, anxiety, depression, catastrophizing, and fear of recurrence until 12 months post-surgery.

**Discussion:**

With perioperative e-CBT targeting preoperative anxiety and pain catastrophizing, we expect to reduce the prevalence and intensity of PPBCT. By means of QST and CPM, we aim to unravel underlying pathophysiological mechanisms. The online application facilitates accessibility and feasibility in a for breast cancer patients emotionally and physically burdened time period.

**Trial registration:**

NTR NL9132, registered December 16 2020.

## Administrative information

Note: the numbers in curly brackets in this protocol refer to SPIRIT checklist item numbers. The order of the items has been modified to group similar items (see http://www.equator-network.org/reporting-guidelines/spirit-2013-statement-defining-standard-protocol-items-for-clinical-trials/).

**Table:** 

Title {1}	AMAZONE: Prevention of persistent pain after breast cancer treatment by online cognitive behavioural therapy- study protocol of a randomized controlled multicenter trial
Trial registration {2a and 2b}.	Netherlands Trial Registry NL9132
Protocol version {3}	V4-amendement-2 / 2022-02-21
Funding {4}	Pink Ribbon / KWF: Dutch cancer Society ESAIC: European Society of Anaesthesiology and Intensive Care
Author details {5a}	Anne Lukas: Department of Anesthesiology & Pain Medicine, Maastricht University Medical Center+ Maurice Theunissen: Department of Anesthesiology & Pain Medicine, Maastricht University Medical Center+ & Department of Clinical Psychological Science, Maastricht University. Dianne de Korte-de Boer: Department of Anesthesiology & Pain Medicine, Maastricht University Medical Center+ Sander M.J. van Kuijk: Department of Clinical Epidemiology and Medical Technology, Maastricht University Medical Center+ Lotte Van Noyen: Department of Clinical Psychological Science, Maastricht University. Walter Magerl: Department of Neurophysiology, Mannheim Center for Translational Neuroscience (MCTN), Ruprecht-Karls-University Heidelberg, Medical Faculty Mannheim Werner H. Mess: Department of Clinical Neurophysiology, Maastricht University Medical Center+ Wolfgang F. Buhre: Department of Anesthesiology & Pain Medicine, Maastricht University Medical Center+ Madelon L. Peters, Department of Clinical Psychological Science, Maastricht University.
Name and contact information for the trial sponsor {5b}	Maastricht University, Faculty of Psychology and Neuroscience, Section Experimental Health Psychology. Madelon.peters@maastrichtuniversity.nl
Role of sponsor {5c}	The study sponsor (Maastricht University) is responsible for study design, data management, data analyses, writing and submitting the report for publication. The study funders (KWF, ESAIC) were involved in study design, and dissemination of the study and its results.

## Introduction

### Background and rationale {6a}

Persistent pain after breast cancer treatment (PPBCT) is highly prevalent [1]. In the acute phase following surgery, reconstructive surgery, radiotherapy, and chemotherapy, up to 50% of the patients suffer from pain [2, 3]. In around 30% (15–65%) of patients, this pain persists for 1 year or longer. Previously, it was thought that pain after mastectomy was mainly neuropathic, caused by damage to the intercostal brachial nerve (ICBN). However, pain may also originate from other sources such as lymphedema, scarring of tissues of the chest wall [4], or from muscle guarding [5]. Once chronic, PPBCT and secondary problems are difficult to treat and require a complex individualized multidisciplinary approach [6, 7]. The standard pharmacological treatment of PPBCT consists of antidepressants [8, 9], and capsaicin [10], but even though effective, intolerable side effects often limit (long-term) treatment. Interventional treatment options of PPBCT target the thoracic dorsal root ganglia, intercostal nerves [11], stellate ganglion [12], thoracic sympathetic chain [13], and fascia of the thoracic wall [14].

In the past decade, research has identified treatment- and patient-related factors increasing the risk for PPBCT. Treatment-related risk factors such as axillary lymph node dissection, radiotherapy, and intercosto-brachial nerve handling may directly increase tissue damage. Patient-related risk factors such as genetic haplotypes, young age, high BMI, pre-existing pain, high postoperative pain intensity, and psychological distress suggest a greater vulnerability of the nociceptive system that is prone to sensitization [15–18]. Quantitative sensory testing (QST) demonstrated the presence of central sensitization in patients with PPBCT [19–22]. Moreover, there is accumulating evidence that patients with persistent postsurgical pain have less efficient inhibitory modulation of afferent nociceptive signals [23–26].

Many interventions aiming at a reduction of nociceptive input and concomitant sensitization of the central nociceptive system have been studied, e.g., reducing the extent of surgery, aggressively treating perioperative pain and minimizing the radiation dose and field [2, 27]. So far, only perioperative ketamine and lidocaine have been found to significantly reduce chronic pain after various kinds of surgery [28]. Perioperative venlafaxine reduced the prevalence of PPBCT in one study [29]. The utility of paravertebral blocks during mastectomy in preventing long-term pain has been studied repeatedly with—despite good acute postoperative pain relief—no convincing preventive effect on PPBCT prevalence [30–36].

Anxiety and pain catastrophizing have emerged as the most robust psychological predictors of persisting postoperative pain [3, 15, 37, 38], including PPBCT [16]. Moreover, these psychological states have been shown to interfere with pain processing within the CNS [39–42]. Stress may alter the endogenous pain inhibition-facilitation balance, resulting in reduced net endogenous pain inhibition [43, 44]. Less endogenous pain inhibition increases the risk for central sensitization and leads to chronification of acute pain.

In contrast to most other identified risk factors for PPBCT, psychological variables are modifiable and can therefore be a target for intervention. Cognitive behavioral therapy (CBT) is the leading psychological treatment for chronic pain. It aims to reduce maladaptive cognitions and behaviors and replace these with more adaptive ones. Reducing anxiety and catastrophizing around the time of surgery may reduce prevalence of persistent pain [45, 46]. A recent meta-analysis showed that perioperative psychological interventions significantly reduced persistent pain and disability after different types of surgeries [47]. The search only identified two RCTs examining the effects on long-term pain after breast cancer surgery. Hadlandsmyth et al. found a 2-h postoperative psychological intervention to be feasible and acceptable, yet it did not significantly affect pain at three months [48]. A one-session preoperative internet-based intervention targeting pain catastrophizing before breast cancer surgery reduced the duration of postoperative of opioid consumption, yet no effect in pain intensity was found [49]. It should be noted that both studies used a single session intervention, which may not be sufficient to have a long-term impact on PPBCT. Moreover, neither study examined the effects beyond the 3-month period.

The AMAZONE study is the first to examine the long-term effects of a more intensive perioperative CBT program on the development of PPBCT in breast cancer surgery patients with high levels of anxiety and/or catastrophizing. Face-to-face CBT is challenging in the context of cancer treatment because of the demands it poses on patients, especially during treatment. Therefore, an online CBT (e-CBT) program was developed. e-CBT increases feasibility because it can be administered in the home environment and at a time that is convenient for patients. It has been demonstrated that (therapist-guided) e-CBT is as effective as face-to-face CBT [50]. A review supported feasibility and acceptability of internet-based interventions for breast cancer patients [51].

In addition to studying the effectiveness of the e-CBT intervention on reducing the prevalence of PPBCT, its effect on sensory (nociceptive) signal transmission and endogenous pain suppressing pathways is examined by means of quantitative sensory testing (QST) and conditioned pain modulation (CPM) to unravel potential underlying mechanisms.

### Objectives {7}

The primary objective is as follows: the primary aim of the project is to investigate the effect of online cognitive behavioral therapy (e-CBT) on the prevalence of PPBCT 6 months after breast cancer surgery in patients with high levels of preoperative anxiety or pain catastrophizing. The e-CBT intervention will be compared with an educational intervention consisting of information about surgery and a healthy lifestyle.

The secondary objective(s) is as follows: to examine the effects of e-CBT on nociceptive sensory signal transmission and central pain inhibiting mechanisms; to examine the effects of e-CBT on the intensity of acute postoperative pain in the week after surgery, cessation of postoperative opioid use, and on PPBCT prevalence after 12 months, PPBCT intensity and interference; and to evaluate the impact of e-CBT on anxiety, depression, catastrophizing, fear of cancer recurrence, and quality of life until 12 months post-surgery.

### Trial design {8}

**Fig. 1:**
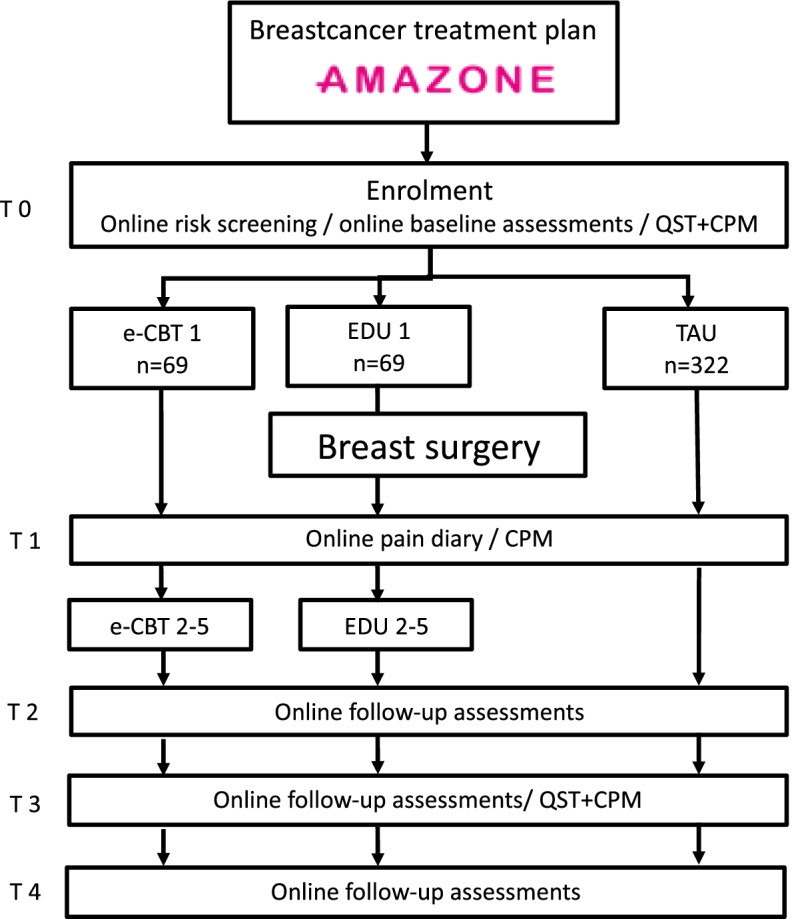
Time schedule of enrolment. T0: 1–3 weeks pre-surgery, T1: postoperative week*, T2: 2 months follow-up, T3: 6 months follow-up, T4: 12 months follow-up. e-CBT, online cognitive behavioral therapy; EDU, online educational therapy; TAU, treatment as usual; QST, quantitative sensory testing; CPM, conditioned pain modulation

This superiority trial is designed as a multi-center randomized controlled trial, with an additional non-randomized control arm. Breast cancer patients with high anxiety or catastrophizing levels will be allocated to either the e-CBT intervention or active control arm (EDU). Patients with low to normal levels of anxiety and catastrophizing will receive treatment as usual (TAU). The TAU group serves as an observational cohort and is followed parallel to the randomized trial (Fig. 1).

## Methods: participants, interventions, and outcomes

### Study setting {9}

Patients are recruited in several academic and specialized hospitals in the Netherlands all dedicated to the treatment of breast cancer.

### Eligibility criteria {10}

The eligibility criteria are as follows: women scheduled for primary breast cancer surgery with or without primary reconstructive surgery.

### Inclusion criteria all patients

- Unilateral primary breast cancer surgery
- Age ≥ 18 years old

### Inclusion criteria for the RCT (e-CBT&EDU)

- Women scoring either ≥ 8 on the anxiety subscale of the Hospital Anxiety and Depression Scale (HADSa) [52], ≥ 3 on the Surgical fear item (i.e., “quite a bit” or “very much” [16];), ≥ 5 on the Concerns about Recurrence Scale (CARS) [53], or ≥ 18 on the Pain Catastrophizing Scale (PCS) [54]

### Inclusion criteria for the observational cohort (TAU)

- Women scoring < 8 on the HADSa, < 3 on the Surgical fear item, < 5 on the CARS, and < 18 on the PCS

### Exclusion criteria all patients

- Breast cancer surgery/treatment in the past
- Preventive breast surgery
- Bilateral surgery
- Not able to understand Dutch
- No internet access
- Visual or hearing impairments interfering with reading and listening to the online material
- Known or suspected severe psychiatric disorder
- Current litigation procedure

### Who will take informed consent? {26a}

Patients are asked for participation by the local breast cancer teams, study nurses, and anesthesiologists involved in the perioperative breast cancer surgery procedure.

If patients are interested, they are offered verbal and written information followed by an appropriate reflection time of 48 h to 1 week, depending on the time-span between preoperative screening and surgery. In adaption to the COVID-19 pandemic, the informed consent procedure is also possible by telephone, video call, mail, and email.

### Additional consent provisions for collection and use of participant data and biological specimens {26b}

Separate informed consent is asked for the use of participant data for future research on breast cancer.

No biological specimens are taken.

## Interventions

### Explanation for the choice of comparators {6b}

To provide a control group with comparable intervention conditions to the e-CBT group, an online educational (sham) intervention is composed. Women randomized to this EDU group receive an intervention based on information about surgery, communication skills, and a healthy lifestyle. Content referring to psychosocial support is avoided. The number of online sessions and appointments with a therapist are equal to the e-CBT intervention.

Women with low to normal levels of anxiety and catastrophizing are allocated to the non-randomized control group receiving TAU. This group serves as reference group for the primary outcome PPCBT and for the QST/CPM measurements.

### Intervention description {11a}

#### AMAZONE e-CBT intervention

The AMAZONE e-CBT intervention is developed together with an experienced onco-psychologist and patient representatives of the Dutch Breast cancer Society and the MUMC+ patient panel. In addition, existing protocols for decreasing pre-operative anxiety and pain catastrophizing [48, 55–57] and fear of cancer recurrence (FCR) [58] were taken into account.

The intervention consists of education and skills training to better cope with pain after surgery and challenge and replace anxious and catastrophizing thoughts by more helpful cognitions. The key elements of the e-CBT are cognitive restructuring, relaxation exercises, coping with anxiety, activity-rest balance, and pleasant activity scheduling. As such, the intervention targets cognitive, emotional, behavioral, and physiological aspects of psychological distress.

The e-CBT intervention consists of five sessions, with one being delivered pre- and four post-operatively. Patients can follow the sessions at their own pace, but are recommended to follow the timeline as presented in Table 1. In addition to the five online sessions, three appointments with a therapist are scheduled. The appointments take place via video call (secured platform). The content is manualized and the duration is limited to 30 min. The purpose of the appointments is to monitor the intervention, increase motivation, and answer questions and concerns that occur during the intervention.

**Table 1:** Summary of the e-CBT intervention

**1st appointment with the therapist:** introduction study intervention and e-CBT platform	
**Session 1: **1–2 weeks before surgery - Education: pain and factors contributing to pain experience; stress response - Relaxation exercises: progressive muscle relaxation, breathing exercises, visualization exercise	
**Day of surgery**	
**Session 2: **DoS + 7 days - Education: relationship between thoughts-feelings-bodily sensations-behavior; coping with anxiety; valued activities - Exercises: thoughts and activity diary	
**2nd appointment with the therapist:** introduction relaxation exercises and thoughts diary; discussion potentially hindering factors	
**Session 3: **DoS + 14 days - Education: different coping strategies; unhelpful and helpful thoughts - Exercises: cognitive restructuring	
**Session 4: **DoS + 21 days - Education: role of avoidance - Exercises: clarifying valued activities; SMART description of new valued activities	
**Session 5: **DoS + 28 days - Recap session 1-4 - Action plan: warning signs and helpful exercises and techniques	
**3rd appointment with the therapist:** discussion on helping thoughts, valuable activities, action plan; evaluation of most helpful elements of the program and how to continue	

*DoS* day of surgery, *SMART* Specific-Meaningful-Acceptable-Realistic-Time framed

Session 1 starts with discussing the reactions that can be expected when confronted with breast cancer and the upcoming surgery. The education part is devoted to pain, the different factors contributing to the pain experience, and the stress response. Relaxation techniques are introduced. Patients are asked to try out the different relaxation exercises (progressive muscle relaxation, breathing exercises and visualization exercise). As a recurring aspect of the intervention, patients choose one exercise and continue to practice this exercise on a daily basis.

Session 2 consists of three parts, the first being the relation between thoughts-feelings-bodily sensations-behavior. With the aid of a thoughts diary, patients learn to notice their own thoughts and reactions. Later in the intervention, this will serve to practice cognitive restructuring of the catastrophizing thoughts. The second part focuses on coping with anxiety and contains tips and exercises. The last part of the session concentrates on finding a good activity-rest balance after the surgery. Patients are encouraged to engage in pleasant or valued activities. After session 2, patients are asked to continue the relaxation exercises, write down their thoughts about cancer, pain, and other complaints in the thoughts diary, and document their pleasant and valued activities.

Session 3 starts with getting insight in one’s own coping strategies. The education part focuses on cognitive restructuring. Patients receive exercises to change their unhelpful (catastrophizing) thoughts into more helpful thoughts. After session 3, patients continue the homework assignments, complemented by the cognitive restructuring part in the thoughts diary.

Sessions 4 is devoted to coping strategies and valued activities. The role of avoidance in the persistence of complaints is discussed. It is explained how valued activities can counterbalance difficult and stressful situations. Therefore, patients are asked to think about things that are genuinely important to them during this period. Using the acronym SMART (Specific-Meaningful-Acceptable-Realistic-Time framed), they describe activities that give them energy and help them endure the difficult treatment. Homework assignments are continued.

Session 5 is devoted to rehearsal, continued practice, and maintenance. After a recap of the first four sessions, patients are guided into the construction of their own action plan. They write down how to recognize their own alarm signals and which of the exercises and techniques from the intervention are most helpful to them.

The intervention is hosted on a specialized eHealth platform (Karify®, Utrecht, Netherlands), which allows secured communication with a therapist.

#### AMAZONE active control intervention (EDU)

The active control intervention (EDU) consists of five sessions that present information that is taken from publicly available sources. It has the same outline as the e-CBT intervention: one online session pre-operatively and four post-operatively and three appointments with a therapist to monitor the intervention, increase motivation, and address possible questions concerning the intervention. The control intervention is hosted on Qualtrics (Qualtrics, Provo, Utah, USA).

Session 1 gives information about the different types of surgery and pain treatment. Patients receive tips on how to prepare for surgery and handle possible complaints the days after surgery. This comprises a series of physical exercises that are recommended after breast cancer surgery. For the patients who want to gain additional information, a list of reliable sources is presented.

Session 2 is devoted to good communication about the disease and treatment.

Session 3 gives information about fatigue during cancer treatment. Different causes are discussed as well as tips on how to cope with fatigue.

Session 4 focuses on a healthy weight and food pattern. Patients can choose the topic(s) they want to read more about: unwanted weight gain, diminished appetite, changed taste, and information on food and medication.

Session 5 consists mainly of rehearsal of the main points of previous sessions.

### Criteria for discontinuing or modifying allocated interventions {11b}

Subjects can leave the study at any time for any reason if they wish to do so without any consequences. The responsible physician or the investigator can decide to withdraw a subject from the study for urgent medical reasons.

### Strategies to improve adherence to interventions {11c}

After enrollment and before the first online session, the randomized patients have an online appointment (via secured platform) to explain the aim of the program and address concerns. Also, after sessions two and five, patients have appointments with their therapists to discuss questions regarding the online sessions and motivate them. This appointment is also meant to create therapeutic alliance and protocol adherence.

When patients forget to fill in the online questionnaires or open the online sessions, they receive reminders via mail or SMS. Patients can also contact the research team if needed.

### Relevant concomitant care permitted or prohibited during the trial {11d}

AMAZONE does not interfere with the planned oncological treatment nor prohibits any other necessary medical, psychological, or psychiatric treatment. In case additional psychological or psychiatric treatment is initiated during the 2 months after surgery in the randomized groups, a sensitivity analysis will be applied to assess potential confounding by this subgroup.

### Provisions for post-trial care {30}

The AMAZONE trial is qualified as a low risk study. Harm from e-CBT or EDU is not expected.

### Outcomes {12}

#### Main study parameter/endpoint

The main outcome of the study is the prevalence of significant PPBCT in the operated area at six months, defined as a score ≥ 3 on an 11-point numeric rating scale (NRS).

#### Secondary study parameters/endpoints

Secondary outcomes of the study are pain intensity scores (intercept and slope) during the first postoperative week (NRS, pain diary), cessation of postoperative opioid use (no. of days), and PPBCT prevalence after 12 months. Mean PPBCT intensity in the operated area (NRS), presence of neuropathic pain (Doleur neuropathique (DN4)) [59], and pain interference (Brief Pain Inventory (BPI) [60], measured at 2, 6, and 12 months.

(Pain) sensitivity is assessed with quantitative sensory testing (QST) before and at 6 months post-surgery. QST is measured at the bilateral pre-axillary dermatomes Th 3. In patients with persistent postoperative pain in the operated area, the 6-month QST measurement is performed in the painful and the corresponding contralateral location. QST measurements are performed according to the protocol developed by the DFNS [61]. Conditioned pain modulation (CPM) is measured before, at 1 week postoperatively and 6 months later according to the suggestions of Yarnitsky et al. [62]. The chosen CPM algorithm compares three repetitions of pressure pain threshold measurements in the thenar contralateral to the operated side, before and immediately after a cold pressor test delivered to the other hand with ice water.

In addition the psychological parameters anxiety, fear of recurrence, catastrophizing, and depression are assessed together with the cancer related quality of life before and up until 12 months after surgery.

#### Other study parameters

Other parameters including clinical and psychosocial patient characteristics, breast-cancer treatment related variables, and compliance with the psychological intervention are assessed at the time points shown in Table 2.

**Table 2:** Parameters and assessment time points

Parameters	Instrument	T 0	T1	T2	T3	T4
Pain operated breast	NRS	x	x	x	x	x
	Pain localization	x		x	x	x
	BPI [60]	x		x	x	x
	DN4 [59]	x	x	x	x	x
Pain unrelated to BC treatment elsewhere	NRS	x		x	x	x
	Localization	x		x	x	x
Shoulder function	DASH [63]	x			x	x
Pain sensitivity	CPM	x	x		x	
	QST	x			x	
Pain catastrophizing	PCS [54]	x		x	x	x
Anxiety	HADS-A [52]	x		x	x	x
Depression	PROMIS short form v1.0 [64, 65]	x		x	x	x
Quality of life	EORTC-QLQ C30+BC [66]	x			x	x
Surgical fear	SFQ [67] & single item [16]	x				
Fear of recurrence	CARS [68]	x		x	x	x
Breast cancer characteristics	TNM, receptors, histological profile	x			x	
	non-related chronic pain complaints	x			x	x
	pain medication	x	x	x	x	x
	Sedative and antidepressive medication	x	x	x	x	x
Patient characteristics	BMI	x			x	x
	Comorbidity and intoxications	x				x
	Unspecific symptoms	x		x	x	x
Breast cancer treatment details	Type of surgery		x	x	x	x
	ICBN handling		x			
	Breast cancer treatment complications		x	x	x	x
	Radiotherapy			x	x	x
	Chemotherapy	x		x	x	x
	Hormonal therapy	x		x	x	x
Anesthesia	Perioperative regional techniques		x			
	Acute pain day 0–7		x			
Patient compliance with protocol				x		

*T0* 1–3 weeks pre-surgery, *T1* postoperative week, *T2* 2 months follow-up, *T3* 6 months follow-up, *T4* 12 months follow-up. *NRS* numerical rating scale (0=no pain, 10=maximum pain), *BPI* brief pain inventory, *DN4* doleur neuropathique 4 questions, *CPM* conditioned pain modulation, *QST* quantitative sensory testing, *PCS* pain catastrophizing scale, *HADS* hospital anxiety and depression scale, *PROMIS* patient-reported outcomes measurement information system, *EORTC-QLQ* European Organization for Research and Treatment of Cancer Quality of Life Questionnaire, *SFQ* surgical fear questionnaire, *CARS* fear of cancer recurrence scale, *DASH* disability of the arm, shoulder and hand questionnaire, *BMI* body mass index, *ICBN* intercostobrachial nerve

#### Participant timeline {13}

The participant timeline is presented in Fig. 1

#### Sample size {14}

The sample size calculation has been performed for the primary outcome, the prevalence of PPBCT (NRS ≥ 3) at 6 months after surgery for breast cancer. The expected overall prevalence of PPBCT is 30%, but the high anxiety/high catastrophizing group, which we will recruit for the randomized trial, has been shown to have a 2.2 times higher prevalence [15, 16, 69], and we expect that about 30% of all patients will screen positive for anxiety/catastrophizing [16, 55, 70]. To be on the conservative side for our calculation, and in accordance with Burns and Moric, we estimate that the prevalence of PPBCT will be 50% in the high anxiety/catastrophizing group [71] and also that perioperative CBT decreases PPBCT prevalence by 50%.

For the current study, this would mean a reduction of the prevalence in the e-CBT group from 50 to 25%. We need to include 55 patients per group to obtain 80% power to detect this difference, with an alpha of 0.05. To account for a potential drop-out rate of 20%, we will recruit a total of 138 high anxiety/catastrophizing patients. All patients that are considered to have low anxiety/catastrophizing and thus are not randomized will be asked to participate in the cohort study. We expect to include around 322 low anxiety/catastrophizing patients based on an expected 30/70% ratio of high vs low anxiety/catastrophizing.

#### Recruitment {15}

Patients of participating hospitals with medium to high production volumes for breast cancer treatment are offered participation by their treating physicians. In addition, information about AMAZONE is shared on a website [64], on websites offering information about breast cancer treatment in the Netherlands, i.e., The Dutch breast cancer society (BVN), The Dutch Cancer Society (KWF), the Integraal Kankercentrum Nederland (IKNL), and also via social media. Hereby we aim to improve recruitment by patient empowerment.

### Assignment of interventions: allocation

#### Sequence generation {16a}

Allocation is performed using electronic stratified randomization with random permuted block sizes. Stratification factors are axillary dissection and center of inclusion.

#### Concealment mechanism {16b}

Patients in the e-CBT and EDU groups are blinded for the type of intervention. Patients in the TAU group cannot be blinded. Treating physicians have access to allocation information.

#### Implementation {16c}

Patients are enrolled by the treating physician at the participating centers. The allocation sequence is computer-controlled and generated by Castor® EDC.

### Assignment of interventions: blinding

#### Who will be blinded {17a}

Patients are only informed about allocation to either treatment (randomization) or control (TAU). Patients who will be randomized will not be informed about the type of intervention—e-CBT or EDU. The local AMAZONE teams can view the treatment allocation in the online patient administration program. Outcome analyses is performed by an independent statistician who will be blinded for the type of intervention.

#### Procedure for unblinding if needed {17b}

The need for unblinding of participants is very unlikely as side effects of CBT are currently not described and EDU does not contain any information/advices that cannot be found elsewhere.

### Data collection and management

#### Plans for assessment and collection of outcomes {18a}

Detailed information on outcomes is presented in the “Outcomes {12}” section. All investigators are working in accordance with GCP guidelines and are trained in the execution of clinical studies.

The teams of the centers assessing pain sensitivity by QST/CPM were trained to perform the standardized QST protocol according to the regulations of the German Research Network Neuropathic pain (DFNS) by the accredited location at the Center for Biomedicine and Medical Technology Mannheim (CBTM), Ruprecht-Karls-University Heidelberg, Medical Faculty Mannheim.

To maintain the quality of the measurements, refresher trainings are organized on a regular basis.

Preoperative risk profiling and all patient reported outcomes (questionnaires) as well as medical information and SAE reporting are collected online with the cloud-based clinical data management platform Castor® EDC (www.CastorEDC.com).

The members of the local AMAZONE study teams are trained to use the program for study flow logistics (Ldot©), Castor©, screening forms and other questionnaires that have to be completed at the respective time points.

Patients are instructed to use the online software by the local investigators during the inclusion procedure.

#### Plans to promote participant retention and complete follow-up {18b}

At the assessment time points, participants automatically receive reminders by email and text messages to fill in the online questionnaires. Members of the local study team can contact the participant if questionnaires are missing or incomplete. In case online questionnaires were not filled in at one time point, patients still receive notifications at the subsequent follow-up assessment times. All collected data will be used for final analyses, even if a participant does not complete follow-up.

#### Data management {19}

Data handling is according to the Dutch General Data Protection Regulation (AVG) and the Dutch Act on Implementation of the General Data Protection Regulation (UAVG). Data are retrieved and stored according to GCP guidelines in a coded fashion in a protected database (Castor® EDC). Subjects will receive a unique sequential study code that does not include any personal information. The coding key will be password protected and kept in each participating hospital, only accessible by the local study team.

An independent quality officer will monitor the study data according to GCP practice. Monitoring encompasses the verification of informed consent, inclusion and exclusion criteria, source data of the clinical parameters and (S)AE reporting, and will take place at the initiation of the study, after inclusion of 10 patients at a site and after inclusion of the last patient.

#### Confidentiality {27}

Information about potential and enrolled participants is documented in a local trial master file (TMF) that is accessible only to the local AMAZONE team. Signed consent forms are collected in a local file. The forms are saved for 15 years after the end of the trial at the study centers.

#### Plans for collection, laboratory evaluation and storage of biological specimens for genetic or molecular analysis in this trial/future use {33}

See the “Additional consent provisions for collection and use of participant data and biological specimens {26b}”; there will be no biological specimens collected.

### Statistical methods

#### Statistical methods for primary and secondary outcomes {20a}

All analyses will be performed according to the intention-to-treat principle. The main analyses focus on the comparison of the e-CBT and education (EDU) group. Exploratory comparisons concerning prevalence of PPBCT, acute postoperative pain, and pain-sensitivity with the no-intervention (low fear) group will also be made, using similar statistical techniques as described below for the two randomized groups.

#### Primary study parameter(s)

Group differences concerning the prevalence of PPBCT at 6 months follow-up will be reported stratified by treatment allocation. Comparisons between e-CBT and EDU will be made using logistic regression analysis, adjusted for the variables used to stratify randomization. The odds ratio (OR) including 95% CI and *p*-value will be reported.

#### Secondary study parameter(s)

The mean daily postoperative pain scores over the 1-week period will be compared between the groups using linear mixed-effects regression with a random intercept and slope to account for multiple measurements over time within patients. Covariance between random effects will be estimated using an unstructured covariance matrix. Correlation between measurements over time will be modeled using a first order autoregressive structure. Both differences in average pain level over time and differences in trend across time will be assessed.

Time to opioid cessation will be analyzed with the Kaplan-Meier method and compared using the log-rank test.

Mean pain intensity and pain interference at 2, 6, and 12 months will be compared using linear mixed-effects regression with regards to group differences and time course.

Quality of life at 6 and 12 months will be compared between groups using the independent-samples *t*-test.

The effect of surgery on QST and CPM parameters will be analyzed by paired-sample *t*-test on the pre-surgical and 1-week and 6-months post-surgical results. The intervention effect on QST and CPM will be assessed with linear mixed-effects regression at the three time points.

Intervention effects on pain catastrophizing, anxiety, depression and fear of cancer recurrence at two months will be evaluated using the independent-samples *t*-test. With linear mixed-effects, regression differences in level and slope are compared between e-CBT and EDU taking all longitudinal measurements into account.

#### Other study parameters

Other parameters including clinical and psycho-social patient characteristics, breast-cancer treatment related variables, and compliance with the psychological intervention are assessed at the time points shown in Table 2.

#### Interim analyses {21b}

After randomization of 50 patients, the effect size for the primary outcome will be computed to assess whether the assumptions made for the sample size calculation were correct. As the responsible statistician is not authorized to decide about the progress of the study—only to give advice about the sample size—the interim analyses will bear no effect on conclusions of effectiveness, but may be used to recompute the necessary sample size.

#### Methods for additional analyses (e.g., subgroup analyses) {20b}

Additional analyses are not planned.

#### Methods in analysis to handle protocol non-adherence and any statistical methods to handle missing data {20c}

In case of missing data in more than 5% of patients, multiple imputation with fully conditional specification will be used to impute the dataset. The number of imputations will be set to the percentage of incomplete patients, and predictive mean matching will be used to draw values to be imputed from selected donors. The percentage of missing values per variable of interest will be presented as count and percentage.

#### Plans to give access to the full protocol, participant level-data and statistical code {31c}

The trial protocol, anonymized trial data, and statistical codes are available upon request from the corresponding author.

### Oversight and monitoring

#### Composition of the coordinating center and trial steering committee {5d}

The study is coordinated by the AMAZONE study group of the Maastricht University and the Maastricht University Medical Center+. Members of the study group also provide the coordination of centers, recruitment, and follow-up on a day-to-day base. The coordinating group meets at least weekly. Monthly meetings of the coordinating group and the recruiting centers are scheduled and a hotline for practical issues is available. Data management support is provided by the center for data and information management at the Faculty of Health, Medicine and Life Sciences of the Maastricht University (MEMIC).

#### Composition of the data monitoring committee, its role and reporting structure {21a}

A DMC was not deemed necessary because the AMAZONE e-CBT and EDU intervention are low-risk interventions.

#### Adverse event reporting and harms {22}

The investigator reports all SAEs for which the assumption can be made that they are related to the imposed behavioral manner (i.e., e-CBT or EDU) to the participating subject or the procedure(s) they are subjected to (QST/CPM measurements). SAEs are reported within the legal timelines (7/15 days) to the sponsor without undue delay after obtaining knowledge of the events. Exemptions from expedited reporting are SAEs that are known for the indication and treatment of breast cancer or other SAE’s for which the assumption cannot be made that they are related to the study intervention. These non-related SAEs do not have to be reported on an expedited basis but will be fully documented on the SAE-eCRF-page.

The sponsor reports the SAEs through the web portal ToetsingOnline and to the accredited METC that approved the protocol, according to the requested timelines by Dutch law.

#### Frequency and plans for auditing trial conduct {23}

An independent auditor (quality officer) will monitor the trial conduct and accuracy of data collection according to the regulations described under Good Clinical Practice (GCP). The quality officer is independent of the sponsor and free from competing interests. In particular, conduction of the informed consent procedure, application of inclusion and exclusion criteria, and the quality of data collection of the primary endpoints are subject to monitoring. The officer will perform a source data verification of data described in the CRFs to investigate the agreement between source data and study reports. The monitor also evaluates whether (S)AE’s are adequately reported within the time frame as directed by the Dutch law.

Monitoring of the centers will take place at the initiation of the study, after inclusion of 10 patients at a site and after that every 6 months until closing the study.

The AMAZONE trial was rated low risk. Monitoring reports are reviewed by the AMAZONE steering committee and reported to the Ethics Committee if necessary according to the WMO.

#### Plans for communicating important protocol amendments to relevant parties (e.g., trial participants, ethical committees) {25}

Protocol modifications and amendments are communicated directly to the local PIs and METCs. In addition recruitment progress, practical information, news from the trial locations are communicated via the monthly meetings, a monthly newsletter, and the study website [64].

#### Dissemination plans {31a}

Trial results will be communicated to the scientific community via journals and national and international conferences. As the AMAZONE study intervention might have immediate impact on the standard of perioperative breast cancer care, special efforts will be made to disseminate the study results to clinicians and health care providers via journals and websites of the national professional associations. As patient empowerment is one of the aims of the AMAZONE study the study protocol is developed in close cooperation with the Borstkankervereniging Nederland and the Dutch Cancer Society. These organizations also have special attention for the information of patients about the study and its results.

## Discussion

In the past decades, breast cancer treatment has evolved to an individualized treatment based on tumor size, receptor status and mutational status. While cancer-treatment options are discussed extensively, information about persisting pain, psychosocial, and physiotherapeutic support are often neglected in the treatment phase. Specialized therapy for these complaints is not structurally offered and if only after patients developed long-lasting complaints. The same is true for studies investigating the effect of e-health interventions to reduce the physical and psychological burden after cancer treatment [51]. These interventions are usually initiated months or years after the completion of breast-cancer treatment.

The AMAZONE study is unique as the intervention starts before breast cancer surgery with the aim to *prevent* the development of persisting pain and related disability. Consequently, functioning and quality of life in breast cancer survivors can be improved. A preventive intervention might have a much larger impact than treating physical and psychological symptoms after they have occurred. A preventive intervention may therefore also be more (cost)effective.

If e-CBT is proven effective by the trial, the AMAZONE application can easily be introduced in daily clinical practice.

## Trial status

The actual protocol version is V4-amendement-2/2022-02-21. Recruitment started in June 2021. To date, 130 patients have been recruited at six study sites in the Netherlands*. The first patient was included June 21, 2021. Planned termination of inclusion is June 2023.

*Study sites: Antoni van Leeuwenhoek Ziekenhuis – The Netherlands Cancer Institute, Amsterdam; Alexander Monro Breastcancer Hospital, Bilthoven; Reinier de Graaf Hospital, Delft; University Medical Center UMCG Groningen, Radboud University Medical Center, Nijmegen; Maastricht University Medical Center+ (MUMC+), Maastricht.

## Data Availability

A descriptive dataset will be forwarded from Datahub to DataverseNL. The anonymized data of patients who gave consent for further use of their data will become available on request after all planned analyses have been performed.

## Abbreviations

PPBCT: Persistent pain after breast cancer treatment
e-CBT: Online cognitive behavioral therapy
EDU: Online education intervention
TAU: Treatment as usual
QST: Quantitative sensory testing
CPM: Conditioned pain modulation
NTR: Netherlands Trial Registry
KWF: Dutch Cancer Society
ESAIC: European Society of Anesthesiology and Intensive Care
HADS: Hospital anxiety and depression Scale
CARS: Concerns about Recurrence Scale
MUMC+: Maastricht University Medical Center
FCR: Fear of cancer recurrence
SMART: Specific-Meaningful-Acceptable-Realistic-Time framed
NRS: Numeric rating scale
PROMIS: Patient reported outcomes measurement information system
EORTC-QLQ: European organization for research and treatment of cancer quality of life questionnaire
SFQ: Surgical fear questionnaire
CARS: Fear of cancer recurrence scale
DASH: Disability of the arm, shoulder and hand questionnaire
BMI: Body mass index
ICBN: Intercosto-brachial nerve
BVN: Dutch Breast Cancer Society
IKNL: Integraal Kankercentrum Nederland
DFNS: German Research Network Neuropathic pain
AVG: Dutch General Data Protection Regulation
UAVG: Dutch Act on Implementation of the General Data Protection Regulation
GCP: Good Clinical Practice
TMF: Trial master file
OR: Odds ratio
CI: Confidence interval
MEMIC: Center for information and data management, Faculty of Health, Medicine and Life Sciences of the Maastricht University
SAE: Serious adverse event
METC: Medisch-ethische Toetsingcommissie
PI: Principal investigator
